# Metachronous cholangiocarcinoma 13 years post resection of choledochal cyst—is long-term follow-up useful?: a case study and review of the literature

**DOI:** 10.1186/s40792-016-0187-9

**Published:** 2016-06-16

**Authors:** Deanna Wan Jie Ng, Adrian Kah Heng Chiow, Wee Teng Poh, Siong San Tan

**Affiliations:** Yong Loo Lin School of Medicine, National University of Singapore, Singapore, Singapore; Hepatopancreatobiliary Unit, Department of General Surgery, Changi General Hospital, Simei Street 3, Singapore, 529889 Singapore; Department of Pathology, Changi General Hospital, Singapore, Singapore

**Keywords:** Choledochal cyst, Cholangiocarcinoma, Metachronous biliary tract cancer

## Abstract

**Background:**

Patients with congenital choledochal cyst are at risk of the development of hepatobiliary malignancy, with recommended treatment of choledochal cyst being surgical excision. The development of cholangiocarcinoma more than 10 years after excision of choledochal cysts is rare, with less than 21 cases reported in the literature from 1972 to 2014. This is the first reported case of metachronous recurrence after a previously excised adenocarcinoma within a choledochal cyst.

**Case presentation:**

Herein, we review the case of a patient with cholangiocarcinoma arising 13 years post excision of a Todani type 1 choledochal cyst and discuss the theories of carcinogenesis and long-term management of patients with choledochal cysts. The long-term development of a malignancy must be considered in these patients.

**Conclusions:**

Reviewing all published cases to date, regular follow-up post resection did not improve on the resectability and long-term survival of these patients. Patients presenting with symptoms did not prejudice against resectability. Despite curative resection, median survival was dismal. Optimal long-term follow-up strategies for these patients remain to be elucidated.

## Background

There is a significant association between congenital choledochal cysts and the development of hepatobiliary malignancies, with an incidence ranging between 2.5 and 28 % [1, 2].

The complete surgical excision of the choledochal cyst with biliary enteric anastomosis is indicated as this reduces the risk of cholangiocarcinoma to between 0.7 and 5.4 % [3]. The time interval and risk factors for the development of cancer post resection are poorly understood, with only several case reports and small case series published to date.

We report a rare case of a cholangiocarcinoma arising 13 years post excision of a Todani type 1 choledochal cyst and discuss the process of carcinogenesis and the merits of follow-up in these patients.

## Case presentation

**Fig. 1:**
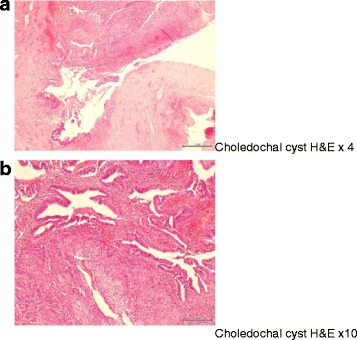
**a** Partly denuded cyst lining with stretches of high-grade dysplasia and invasive tumour at the *upper edge*; see Fig. 2b for ×10 view of area. **b** Invasive adenocarcinoma featuring both invasive irregular glands and solid tumour with clear cytoplasm

A 59-year-old Chinese lady has been on follow-up with our institution for a previous Todani type 1 choledochal cyst for which she underwent cyst excision with Roux-en-Y hepaticojejunostomy in 2001. Subsequent pathology revealed the cyst containing an incidental focus of adenocarcinoma with clear margins of resection (Fig. 1).

**Fig. 2:**
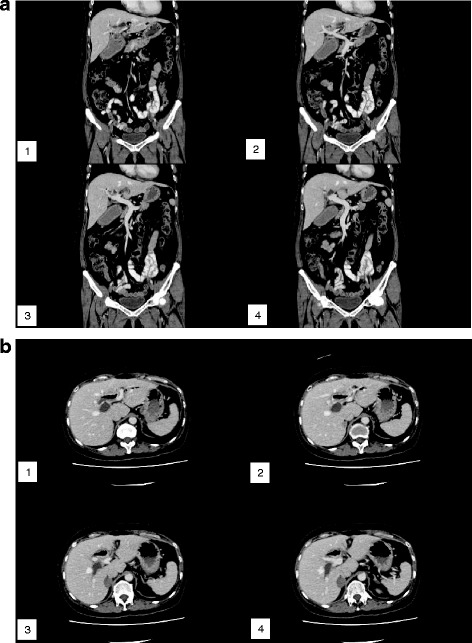
**a** Coronal view of the CT abdomen showing no recurrence of tumour. **b** Transverse views of the CT abdomen showing no recurrence of tumour

Since resection, she had been regularly reviewed with computed tomography (CT) scans of the abdomen and CA 19-9 levels performed annually. A CA 19-9 level performed in 2012 showed a slightly elevated level of 52 IU/ml. Magnetic resonance imaging (MRI) of the liver was subsequently performed in October 2012 did not show any evidence of recurrence. An interval CT abdomen performed in December 2013 (Fig. 2) was similarly unremarkable, and she was given a subsequent appointment for a repeat CA 19-9 and imaging in a years’ time.

**Fig. 3:**
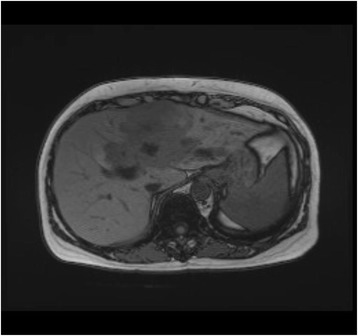
MRI liver showing a central large heterogenous mass inseparable and encasing the main portal vein and biliary ductal confluence suggestive of cholangiocarcinoma

**Fig. 4:**
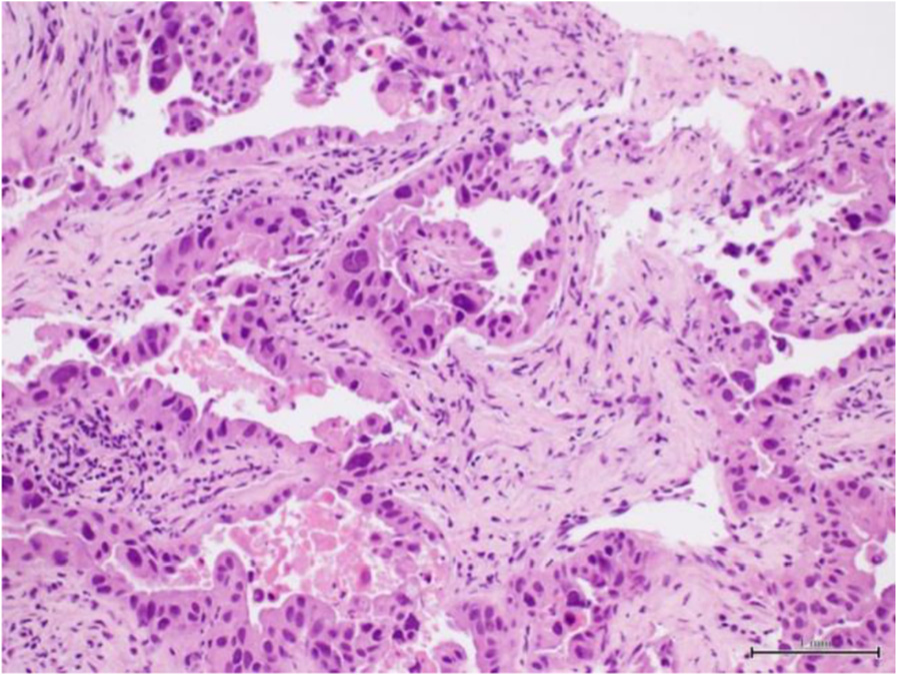
Endoscopic ultrasound-guided biopsy of the mass showing findings of moderately differentiated adenocarcinoma consistent with cholangiocarcinoma

She represented early for clinic review in October 2014 complaining of intermittent fevers for several months. These resolved with short courses of antibiotic therapy prescribed by her local general practitioner. During this period, there was a slight elevation of transaminases and the CA 19-9 level rose to 487 IU/ml. A magnetic resonance cholangiopancreatography (MRCP) was performed which showed a central large heterogenous mass encasing the main portal vein and biliary ductal confluence suggestive of cholangiocarcinoma (Fig. 3). There was also an indeterminate filling defect in the right intrahepatic duct and loss of signal in the middle and left hepatic veins. An endoscopic ultrasound-guided biopsy of the mass confirmed the finding of moderately differentiated adenocarcinoma consistent with cholangiocarcinoma (Fig. 4). A percutaneous trans-hepatic cholangiography and biliary drainage was subsequently performed for worsening biliary obstruction. Bile culture was positive for *E. coli* spp., and amylase levels were normal.

This patient was discussed at the multidisciplinary tumour board, and decision was made for palliative chemotherapy for locally advanced unresectable disease. The patient received 3 cycles of gemcitabine 1300 mg and cisplatin 35 mg. However, this was complicated by recurrent cholangitis and sepsis leading to early cessation of chemotherapy. This eventually led to progressive disease, and the patient passed away 10 months post diagnosis of her recurrence.

## Conclusions

Cholangiocarcinoma is the second most common primary liver cancer after hepatocellular carcinoma and accounts for approximately 10–25 % of all hepatobiliary malignancies [4]. Risk factors for the development of cholangiocarcinoma include parasitic infections, primary sclerosing cholangitis, choledochal cysts, hepatolithiasis, and toxins [5]. The development of cholangiocarcinoma can also occur after biliary enteric anastomoses. Tocchi et al. reported a rate of 5.5 %, where 55 out of 1000 cases developed cholangiocarcinoma after biliary enteric anastomoses for benign pathologies [6].

There have been 31 cases (Table 1) of cholangiocarcinoma occurring after resection of choledochal cyst reported in the literature between 1972 and 2014 with the median time to recurrence being 6 years (range 2–34 years). The most common type of choledochal cysts in these patients was Todani type 4a. Our patient had a recurrence 13 years post resection of a Todani type 1 cyst. In addition, our patient had an incidental focus of adenocarcinoma in the initial cyst. This is the first reported case of metachronous recurrence after a previously excised adenocarcinoma within a choledochal cyst [7–24]. Moertel first proposed a definition for synchronous cancers as those occurring within 6 months of the first primary cancer and for metachronous cancers, as those occurring more than 6 months later. While synchronous gallbladder and biliary tree cancers have been reported between 5 and 7.4 % of cases, occurrence of metachronous cancers has been rarely reported for extra-hepatic bile duct cancers [25].

**Table 1:** Publications of cholangiocarcinoma occurring after resection of choledochal cyst reported in the literature

Study	No. of patients with CC	Median age (years)	Type of cyst	Median duration to development of CC (years)	Median follow-up (months)	Site of cancer	Treatment	Median survival after development of CC (months)
Case reports 1967–2014 [4, 7–21]	18	39 (18–62)	14 type 4a, 2 type 1, 2 ND	18 (2–34)	48 (24–120 from 4 studies)12 studies with no follow-up done	9 intrahepatic duct, 7 hilar, 1 intrapancreatic, 1 ND	9 resected, 4 unresectable, 5 death before surgery	Resected, 10 (6-30), Unresectable 3 (2-4)
Ohashi et al. 2013 [11]	4	41 (27–65)	2 type 1, 2 type 4a	19 (13–32)	181 (7–484)	2 intrahepatic duct, 1 hilar, 1 intrapancreatic duct	All resected	15 (9–43)
Rossi et al. 1986 [16]	6	41 (22–70)	1	4 (1–18)	39	4 extra-hepatic bile duct, 2 intrahepatic bile duct	3 resected, 3 unresectable	4 (1–6)
Scudamore et al. 1994 [22]	1	ND	4 a	2	24	Intrahepatic	Resected	24
Joseph et al. 1996 [23]	1	29	4 a	3	ND^a^	Intrahepatic	Unresectable	36
Shah et al. 2009 [24]	1	42	4 a	2	28	Extra-hepatic bile duct	Unresectable	2

*ND* not documented, *CC* cholangiocarcinoma

Anomalous union of pancreaticobiliary ductal (AUPBD) junction without congenital choledochal cyst is a risk factor for hepatobiliary malignancies and is defined as the abnormal junction of the pancreatic duct and common bile duct that occurs outside the duodenal wall to form a long common channel (>15 mm). Kimura et al. has proposed a classification system for AUPBD where type 1 is defined as the pancreatic duct joined to the common bile duct (as the main duct) and type 2 is defined as the common bile duct joined to the pancreatic duct (as the main duct) [26]. The anomalous junction is also often associated with a choledochal cyst or a biliary tract carcinoma which may be due to the reflux of activated pancreatic fluid; type 1 abnormalities carry a higher risk of biliary tract and gallbladder malignancies [27].

Various theories have been put forward to explain the development of malignancy in these patients with previously resected choledochal cysts. Firstly, some authors have suggested that the epithelium of the remnant bile duct wall is already at a precancerous stage at the time of surgery and hence, the development of cholangiocarcinoma is merely a result of carcinogenesis during the postoperative period [7]. Secondly, the existence of stenosis at the anastomosis or in the intrahepatic bile duct may induce carcinogenesis. Moreover, some have postulated that carcinogenesis is caused by repeated damage of the biliary epithelium by bile fluid as well as bacterial contamination which leads to mucosal metaplasia [8]. Lastly, cholangiocarcinoma can develop spontaneously in the general population which may explain the largely variable intervals of presentation of cholangiocarcinoma in these patients.

Kumamoto et al. have postulated that the reflux of activated pancreatic juice is the strongest contributing factor to carcinogenesis. A study of biliary enteric reconstruction performed for benign disease has shown that the incidence of cholangiocarcinoma after choledochoduodenostomy is higher compared to hepaticojejunostomy (7.6 vs. 1.9 %) [6]. They postulate that this significant difference in incidence rate occurs because activated pancreatic juice can more easily flow back to the biliary tract in a choledochoduodenostomy as compared to a hepaticojejunostomy and hence, the reflux of activated pancreatic juice might be the strongest carcinogenic factor. However, of the cases reported in the literature, few patients had reconstruction with a choledochoduodenostomy, suggesting that unlike other benign pathologies, in patients with previous choledochal cysts, reflux of pancreatic juice may not be the strongest carcinogenic factor.

Other studies have suggested that the epithelium of the remnant bile duct wall is already at a precancerous stage at the time of surgery and hence, development of cholangiocarcinoma is merely a result of carcinogenesis during the postoperative period. This is seen mostly in Todani type 4a cysts where development of cholangiocarcinoma mainly occurs in the dilated intrahepatic bile duct [9]. Our patient developed cholangiocarcinoma after excision of a type 1 cyst, suggesting that despite the initial cyst only involving extra-hepatic ducts, the entire biliary tree despite being of normal calibre may have a high risk of field cancerization.

While the risk of interval malignancy is well described, there are no guidelines for the duration of follow-up and the type of investigations that patients should undergo after initial surgery for choledochal cyst. In the 18 case reports in the literature, 17 patients were not followed up routinely with radiological imaging and tumour markers as compared to our patient who was followed up yearly with CT scans and CA 19-9 levels. Twelve of these patients had no long-term follow-up. Only one patient had regular hepatobiliary system (HBS) ultrasound, tumour markers and liver function test (LFT) in view of her hepatitis C status. In all 17 patients without routine imaging or tumour marker follow-up post excision of choledochal cyst, cholangiocarcinoma was detected only when these patients were symptomatic with cholangitis at presentation. In the one patient with regular follow-up, development of cholangiocarcinoma was detected based on ultrasound HBS.

Nishayama et al. has suggested lifelong biannual follow-up with CT scans and CA 19-9 levels in patients with high risk of development of cholangiocarcinoma and annual ultrasounds in patients with low risk [10]. High-risk features include the presence of dilated biliary ducts post resection and reconstruction with choledochoduodenostomy and hepatitis. The rationale behind such stringent follow-up is that resection would be more difficult or even not be possible in patients with more advanced disease. The belief is that with tighter follow-ups, development of cholangiocarcinoma can be picked up at an early stage, giving these patients a greater chance of cure [8].

Including our patient, 21 out of 32 patients had development of cholangiocarcinoma more than 10 years post excision of choledochal cyst (Table 2). Of these 20 patients, 12 underwent curative resection (60 %). However, 11 patients had no long-term follow-up and recurrences were detected due to the patients’ symptoms. This is in comparison with 5 out of 11 patients with development of cholangiocarcinoma less than 10 years post excision of choledochal cyst that underwent curative resection of cholangiocarcinoma (45 %). Of these five patients, four patients (80 %) were followed up regularly with clinic reviews till recurrence of cholangiocarcinoma. This suggests that perhaps follow-up with regular clinic reviews alone does not affect resectability of cholangiocarcinoma. Also, detection of cholangiocarcinoma based on the patient’s symptoms does not inevitably result in unresectable disease.

**Table 2:** Characteristics of patients (including current study)

	Resected	Unresectable
Cholangiocarcinoma development more than 10 years post excision (*n* = 21)	12	9
Cholangiocarcinoma development less than 10 years post excision (*n* = 11)	5	6

The follow-up of such patients with radiological imaging and blood investigations over their lifetime would expose patients to repeated procedures. This may have been useful to detect recurrences that occur <10 years post resection with 80 % of recurrence detected on follow-up. For detection beyond 10 years post resection, the optimum strategy for follow-up is uncertain. This was seen in our patient where even though tumour markers and imaging were performed annually, the disease was still advanced and unresectable at presentation.

More importantly, this study demonstrates that there is a significant long-term risk of cholangiocarcinoma recurring even after 10 years. These patients should be offered long term regular follow-up with a combination of clinical evaluation, radiological imaging and tumour markers.

In addition, in terms of overall survival, the literature shows overall survival rates after resection of cholangiocarcinoma in these patients to be 50 % at 2 years posttreatment and 25 % at 3 years posttreatment, with a median survival time of 15 months, with no 4-year survivors [11]. This suggests that despite being able to perform curative resection, biliary malignancy shows an unfavourable outcome reflecting a more aggressive biology then de novo cholangiocarcinoma.

In conclusion, this is a case of intrahepatic cholangiocarcinoma arising 13 years after excision of a choledochal cyst. We assessed the theories of carcinogenesis and postulated that the cancer development was related to the reflux of intestinal bacteria into the bile duct. While long-term follow-up should not be discouraged, the available literature suggest that the best strategy remains to be defined due to the variability in the duration of the representation, the advanced disease of representation, and the poor prognosis.

## Consent

### Ethics approval and consent to participate

Ethics approval was obtained from the SingHealth institutional review board for this study, and consent for study and publication was obtained from the next of kin.
